# Effects of ethyl palmitate on the release of aroma compounds in propanediol–ethanol solution and its mechanisms

**DOI:** 10.3389/fchem.2024.1381835

**Published:** 2024-06-10

**Authors:** Wei-Wei Guo, Li-Wen Luo, Ding-Zhong Wang, Ying-Jie Fu, Hui Xi, Shi-Hao Sun

**Affiliations:** ^1^ Flavour Science Research Center, College of Chemistry, Zhengzhou University, Zhengzhou, China; ^2^ Department of Flavor, Zhengzhou Tobacco Research Institute, Zhengzhou, China; ^3^ Food Laboratory of Zhongyuan, Luohe, China; ^4^ Technology Center Laboratory, Jilin Tobacco Industrial Co., Ltd., Changchun, China

**Keywords:** ethyl palmitate, release, kinetics, *Osmanthus fragrans* flower absolute, two-film model

## Abstract

Long-chain esters (LCEs) are known to affect aroma perception, but the mechanism of their effects remains unclear. In this study, ethyl palmitate (EP), an important LCE in *Osmanthus fragrans* flower absolute (OFFA), was selected as a target to identify its role and mechanism. The release characteristics of 10 aroma compounds from OFFA with and without EP were obtained by headspace gas chromatography mass spectrometry (HS-GC/MS) and olfactometry evaluation, respectively. The results show that EP changes the release behaviors of volatile compounds in solution, increases their olfactory detection thresholds (ODTs), and reduces the equilibrium headspace concentrations. According to Whitman’s two-film model, EP was found to change the partition coefficients and mass transfer coefficients of the compounds between the liquid and gas phases. This indicates that EP plays an important role in the scent formation of a flavor product and that it is very valuable for the style design of the flavor product.

## Graphical Abstract

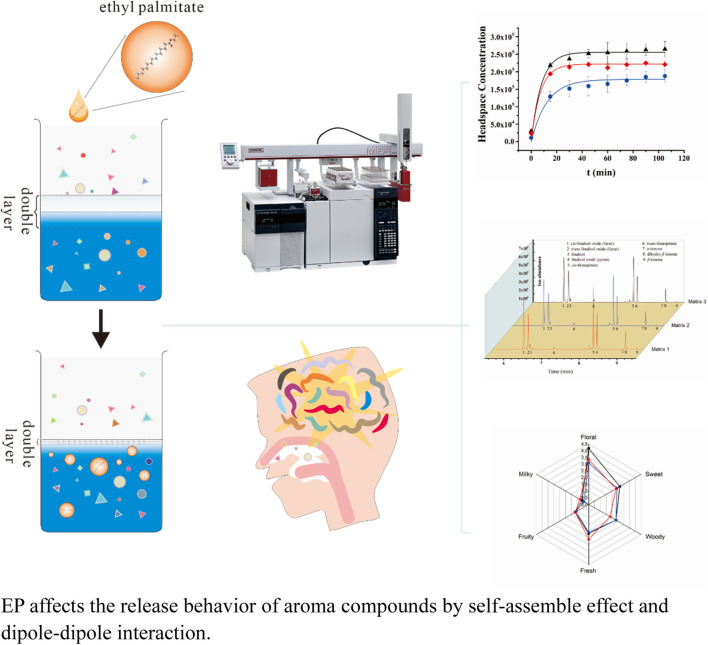

## 1 Introduction

*Osmanthus fragrans* flowers are widely used in perfume production owing to their rich and captivating fragrance (Liu et al., 2022). Therefore, there are extensive research reports in literature on the volatile compounds of Osmanthus (Hu et al., 2009; Hu et al., 2010; Lei et al., 2016). However, understanding only the volatile components in a material is not sufficient to fully predict aroma perception, as this process depends heavily on the interactions between the volatile and non-volatile compounds (Teixeira et al., 2013). It is commonly known that some low-volatility or non-volatile compounds are also important components of *O. fragrans* flower absolute (OFFA) (He and Qi, 2020) and that these account for a relatively high content of around 25% of the components (Qiu, 2000). Long-chain esters (LCEs) usually represent the low-volatility or non-volatile components in OFFA. The relationship between the LCE content and OFFA release behavior is not completely clear. Therefore, investigation of the interactions between LCEs such as ethyl palmitate (EP) and the aroma components in OFFA is crucial for better understanding of the role of Osmanthus aroma in perfumery.

The process of release of aroma components from the bulk to gas phases plays an important role in aroma perception, especially for a homogenous solution. Several researchers have reported the factors influencing the release of aroma compounds (Rabe et al., 2003; Mitropoulou et al., 2011; Lorrain et al., 2013; Villamor et al., 2013; Rahn et al., 2019; Guo et al., 2020; Gabler et al., 2024; Xiao et al., 2024); these factors depend on not only the physicochemical properties but also the influence of low-volatility or non-volatile components in the solution. As a result, the content of low-volatility or non-volatile components in the solution determines the amount of aroma compounds transferred to the headspace, affecting the overall aroma profile (Roussis and Sergianitis, 2008; Cameleyre et al., 2018; Niu et al., 2020).

Recently, some researchers have focused on the LCEs in the flavor system. Boothroyd et al. (2012) reported the influences of LCEs (C6-C16) on volatile partitioning in a whisky model system. Xiao et al. (2019) reported that ethyl tetradecanoate could enhance the floral scent and overall aroma of rose oil. Hsieh et al. (2014) found that EP, ethyl oleate, and ethyl linolenate could significantly alter the sensory profiles of Taiwanese rice spirits. These studies focused on alterations in the release amounts of the aroma compounds, partition coefficients, or olfactory intensities. However, insufficient attention was paid to the changes in the release kinetics. Further research is therefore essential to elucidate why changes in the LCEs affect aroma perception.

The present study aimed to investigate the effects of EP on the release of the main aroma components from OFFA. Accordingly, the releases of 10 aroma compounds in solution were analyzed using headspace gas chromatography mass spectrometry (HS-GC/MS) and subjected to olfactory evaluations at various EP concentrations. The release kinetics of the compounds were discussed on the basis of Whitman’s two-film model.

## 2 Materials and methods

### 2.1 Chemicals and materials

OFFA was purchased from Guangzhou Rihua Flavor & Fragrance Co., Ltd. (Guangzhou, China). All the chemicals (purity >98%) were analytical grade and purchased from various chemical reagent companies as follows: 1,2-propanediol and linalool from J&K Scientific Co., Ltd. (Shanghai, China); ethanol from Shanghai Chemisci Technology Co., Ltd. (Shanghai, China); cyclohexane from CINC High Purity Solvent Co., Ltd. (Shanghai, China); 1-phenylethyl propionate from Adamas Reagent Co., Ltd. (Shanghai, China); 4-methoxyphenethy alcohol and γ-decalactone from TCI Development Co., Ltd. (Shanghai, China); β-ionone, dihydro-β-ionone, phenethyl alcohol, and linalool oxide (mixture of isomers) from Sigma-Aldrich (Shanghai, China); theaspirane (mixture of cis and trans isomers) from Shanghai Titan Scientific Co., Ltd. (Shanghai, China); α-ionone from Shanghai Aladdin Biochemical Technology Co., Ltd. (Shanghai, China); EP from Shanghai Macklin Biochemical Technology Co., Ltd. (Shanghai, China).

Reconstituted oil (RO) was prepared on the basis of the quantitative results of the aroma compounds (shown in Supplementary Table S2) in the OFFA, and the ingredients of the RO are listed in Supplementary Table S3.

### 2.2 GC/MS analysis of OFFA

OFFA was analyzed on an Agilent 8,890 gas chromatography system coupled with a 5977B mass spectrometer and a DB-5MS capillary column (60 m × 0.25 mm × 0.25 μm). The GC oven temperature was first maintained at 50°C for 1 min, immediately followed by ramping at 10 °C/min up to 300°C and then maintaining at 300°C for 10 min. Helium was used as the carrier gas at a constant flow rate of 1.0 mL/min. The transfer line was maintained at 300°C. The mass spectrometer was operated in the electron impact mode (70 eV). The mass was scanned in the range of 50–300 amu. A 0.0100 g solution of 1-phenylethyl propionate in cyclohexane (0.02 mg/g) was added to 1.0000 g of the OFFA cyclohexane solution (40 mg/g). Here, 1-phenylethyl propionate was used as the internal standard. The OFFA solution was used for both qualitative and quantitative analyses. The chromatography peaks were identified by comparing the resulting mass spectra with the NIST database.

### 2.3 Analysis of headspace release of aroma compounds

#### 2.3.1 Sample preparation

Matrix 1 was prepared by mixing ethanol and 1,2-propanediol (v/v, 1:1). Matrix 2 and Matrix 3 were obtained by adding 0.5% and 1% (v/v) EP to Matrix 1, respectively. The aroma compounds and RO were then added sequentially to Matrix 1, Matrix 2, and Matrix 3 to prepare a series of 10 mg/g solutions. These solutions were next analyzed by HS-GC/MS.

#### 2.3.2 HS-GC/MS analysis

Static headspace analysis of each solution was conducted as follows: 1 mL of the solution was carefully added to a 20 mL vial at room temperature (25°C ± 1°C). After fast sealing and equilibration for 2 h, 1 mL of the headspace gas was retrieved for GC/MS analysis. The headspace sampling was conducted intermittently using the Gerstel multiple automatic sampler system (Mülheim an der Ruhr, Germany). Dynamic headspace analysis of each solution was conducted as follows: 1 mL of the solution was carefully added to a 20 mL vial at room temperature; after rapid sealing, 1 mL of the headspace gas was retrieved every 15 min for GC/MS analysis.

Both the dynamic and static headspace analyses used consistent GC/MS parameters. The GC/MS analyses were performed on an Agilent 8,890 gas chromatography system coupled with a 5977B mass spectrometer and a DB-5MS capillary column (60 m × 0.25 mm × 0.25 μm). The oven temperature was programmed to maintain 50°C for 1 min, then increase to 250°C at the rate of 20 °C/min, followed by holding for 2 min. Helium was used as the carrier gas at a constant flow rate of 2.18 mL/min. The mass spectrometer was operated in the electron impact mode (70 eV). The mass was scanned in the range of 50–300 amu. The chromatographic peaks of each compound were recorded as the headspace concentrations (HCs) of its volatile components. All data were obtained with five repetitions per sample.

### 2.4 Sensory analysis

The olfactory detection thresholds (ODT) of each of the compounds in different matrices were evaluated using the three-alternative forced choice (3-AFC) and best estimation threshold (BET) methods (ISO-13001, 2018). Seven volunteers (four females and three males, aged 22–28 years, with expertise in food science or sensory science) from the Laboratory of Aroma Research Center, College of Chemistry, Zhengzhou University, participated in this study. Before the experiments, the volunteers were trained for 3 months on sensory evaluations. All participants provided informed consent prior to participation. All study procedures were approved by the Aroma Research Center, College of Chemistry, Zhengzhou University.

The initial concentration of each flavor component in the different matrices was 10 mg/g and was serially diluted tenfold thereafter. For each solution concentration, the panelists were requested to distinguish the sample containing the volatile compound along with two blank contrast samples. The BET was calculated as the geometric mean of the highest concentration corresponding to the erroneous selection and its adjacent higher concentrations. The group BET was then calculated as the geometric mean of the individual BET values.

The seven panelists determined the characteristic aroma descriptors of the RO, and the aroma profile of the RO was subsequently assessed on the basis of the selected aroma descriptors.

### 2.5 Dipole moment

Theoretical calculations were conducted using the Gaussian 09 software. The structures of the aromatic compounds and EP were optimized at the theoretical level of M06-2X/6-31G (d, p) under the solvation model based on density (SMD) (Marenich et al., 2009) model, and the dipole moments were calculated subsequently.

## 3 Results and discussion

### 3.1 Main chemical composition of OFFA

The OFFA sample was analyzed by GC/MS (Supplementary Figure S1), and a total of 29 compounds (summarized in Supplementary Table S2) were identified using the NIST mass spectra database; these compounds contributed to 97% of the total chromatographic effluent.

About 38% of the total chromatographic peak area of OFFA entailed long-chain compounds, including EP (3.94%), palmitic acid (5.30%), and (*Z,Z,Z*)-9,12,15-octadecatrienoic acid (12.15%); these were low-volatility or non-volatile compounds with weak or no odors. Some studies have reported that the non-volatile compounds could influence the release of aroma compounds in flavor products [10]. Preliminary experiments showed that the addition of LCEs (such as EP) to OFFA could significantly change the headspace composition and aroma profile of the oil (results shown in Supplementary Figure S2). Consequently, further experiments were conducted to investigate the impacts of the LCEs on the release of the main aroma compounds in OFFA.

### 3.2 Effects of EP on the release of aroma compounds

Ten aroma compounds from OFFA were selected as the targets to evaluate the influence of EP on the release of aroma components, including linalool oxide (furan enantiomers), linalool oxide (pyran), dihydro-*β*-ionone, *α*-ionone, *β*-ionone, *γ*-decalactone, theaspirane (enantiomer), and linalool. These have always been considered as some of the main contributors to the characteristic odor of OFFA (Xin et al., 2013).

#### 3.2.1 Selection of solvent for release of aroma compounds

Previously, 1,2-propanediol was considered as a suitable solvent for aroma compounds in experiments related to aroma release because of its low vapor pressure, which avoided competition between the flavors and solvent in the release procedure from liquid phase to the headspace (Reineccius et al., 2005; Yang et al., 2013). However, the poor solubility of EP in 1,2-propanediol did not meet the requirements of the gradient experiments. Compared with 1,2-propanediol, ethanol as a commonly used solvent in perfumes (Miastkowska et al., 2018) is more suitable for the solubility of ester compounds; however, the high vapor pressure of ethanol could affect the results. For our purposes, a mixture of propylene glycol and ethanol, which is commonly used as a solvent for fragrance, was considered as the solvent to solve the dilemma (Guo et al., 2019).

**FIGURE 1:**
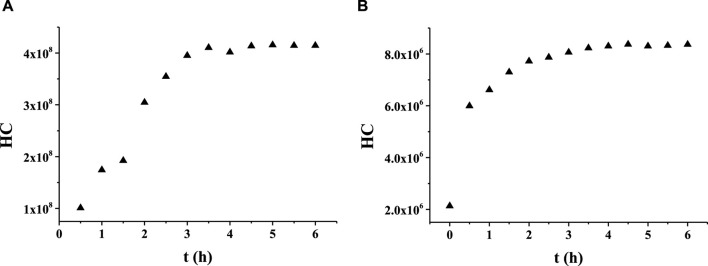
Release behaviors of linalool in **(A)** 1,2-propanediol and **(B)** the mixed solution of 1,2-propanediol and ethanol.

A series of experiments was conducted to optimize the ratio of 1,2-propanediol to ethanol in the solvent. A mixture of 1,2-propanediol and ethanol was first prepared in the volume ratio of 1:1, which was denoted as Matrix 1. The solubility test revealed that EP dissolved completely in Matrix 1 in the range of 0.5%–10% (v/v). Solutions of linalool at concentrations of 10 mg/g each were prepared in both 1,2-propanediol and Matrix 1 for a comparative study of the release behaviors. Subsequently, the two samples were analyzed by HS-GC/MS to measure the time-related HCs of linalool (Figure 1). The results indicated that the release behavior of the aroma compounds in the mixed solvent was similar to that in 1,2-propanediol. Thus, the mixture of 1,2-propanediol and ethanol was selected as the solvent for the subsequent release experiments.

#### 3.2.2 Effects of EP on the release process of aroma compounds

Each compound was added separately to Matrix 1, Matrix 2, and Matrix 3 to prepare solutions at a concentration of 10 mg/g. The corresponding HCs were measured by HS-GC-MS. Many studies have proposed that the release process of volatile components in a homogeneous solution could be explained by Whitman’s two-film model (Whitman, 1962; Banavara et al., 2002; Hu and Chai, 2013). This model assumes the existence of a double layer at the liquid–gas interface, comprising a liquid phase membrane and a gas phase membrane. The mass transfer of volatiles across the interface is considered to be proportional to the concentration difference of the volatiles between the liquid and gas membranes. According to the model, the mass transfer process can be expressed by Eq. (1), which can be integrated within appropriate limits to derive Eq. (2).

$$V\left(d\;C_{hs}/dt\right)=KA\left(K_{\mathrm{lg}}C_{bp}‐C_{hs}\right)$$ (1)

$$C_{hs}\left(t\right)=K_{\mathrm{lg}}C_{bp}\left(1‐\exp\;\left[-\right.\left(\frac{KA}{V}\right)t\right)$$ (2)

Here, V is the volume of the gas phase in the sample bottle, C_hs_ is the HC of the volatile compound, t is the release time, K is the mass transfer coefficient, A is the area of the liquid–gas interface, K_lg_ is the partition coefficient of the volatile compound in the liquid–gas phase, and C_bp_ is the amount of volatile substance in the liquid phase.

**FIGURE 2:**
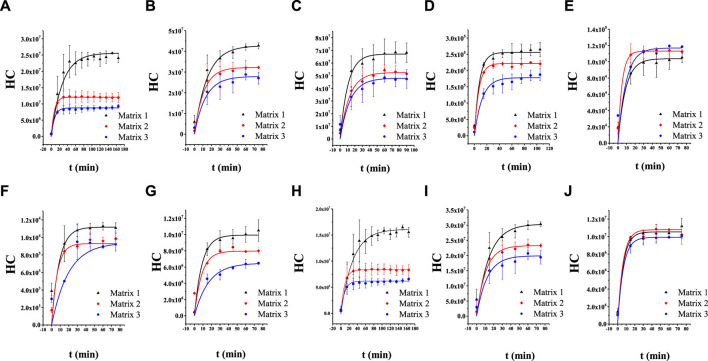
Release curves of the aroma compounds in Matrix 1, Matrix 2, and Matrix 3: **(A)** *cis*-theaspirane; **(B)** *cis*-linalool oxide (furan); **(C)** linalool oxide (pyran); **(D)** α-ionone; **(E)** *β*-ionone; **(F)** *γ*-decalactone; **(G)** dihydro-*β*-ionone; **(H)** *trans*-theaspirane; **(I)** *trans*-linalool oxide (furan); **(J)** linalool.

The parameters C_bp_, K, A, K_lg_, and V are constant for a specific system; this means that the HCs of the aroma compounds are logarithmically related to the release time. The dynamic HC of each compound was obtained and plotted as the HC–time curve (Figure 2, and the corresponding fitting equations are provided in Supplementary Figures S3-S12; the curves are plotted using the function BoxLucas 1 in Origin Pro 9. As expected, the curves exhibit the classical characteristics of the logarithmic function. These results show that the release process of aroma compounds in Matrix 1, Matrix 2, and Matrix 3 could be expressed by the two-film model. The values of the parameters, including HC, K, and determination coefficient (R^2^), were obtained according to the model and are summarized in Table 1. The addition of EP changed the K values of all these compounds except for linalool. According to Eq. (1), the change in K value leads to a change in the release rate. Therefore, the addition of EP could change the mass transfer process of the volatiles reaching liquid–gas distribution equilibrium.

**TABLE 1:** Headspace concentrations (HCs), mass transfer coefficients (K), and determination coefficients (R^2^) of 10 aroma compounds based on Whitman’s two-film model.

Aroma compound	Matrix 1			Matrix 2			Matrix 3		
	HC	K	R^2^	HC	K	R^2^	HC	K	R^2^
*β*-Ionone	103,788	0.113	0.88	1,130,098	0.212	0.94	116,984	0.107	0.72
*α*-Ionone	255,786	0.126	0.80	221,898	0.138	0.98	178,296	0.081	0.89
*γ*-Decalactone	11,169	0.114	0.60	9,290	0.151	0.91	9,395	0.051	0.88
Dihydro-*β*-ionone	9.93 × 10^6^	0.113	0.98	7.96 × 10^6^	0.117	0.72	6.49 × 10^6^	0.060	0.98
*cis*-Linalool oxide (furan)	4.27 × 10^7^	0.067	0.96	3.22 × 10^7^	0.091	0.99	2.79 × 10^7^	0.076	0.96
*trans*-Linalool oxide (furan)	3.04 × 10^7^	0.068	0.92	2.38 × 10^7^	0.066	0.99	1.99 × 10^7^	0.082	0.93
Linalool oxide (pyran)	6.71 × 10^7^	0.096	0.67	5.36 × 10^7^	0.079	0.98	4.78 × 10^7^	0.074	0.86
*cis*-Theaspirane	2.56 × 10^7^	0.036	0.99	1.21 × 10^7^	0.135	0.96	8.77 × 10^6^	0.124	0.97
*trans*-Theaspirane	1.61 × 10^7^	0.034	0.98	8.35 × 10^6^	0.113	0.97	6.16 × 10^6^	0.113	0.96
Linalool	1.05 × 10^7^	0.157	0.95	1.07 × 10^7^	0.163	0.96	9.91 × 10^6^	0.153	0.97

The mass transfer coefficient is expressed as K = D/δ in the model, where D is the diffusion coefficient, and δ is the thickness of the double layer (Danckwerts and Kennedy, 1997). According to the Stokes–Einstein law, D is inversely proportional to the viscosity of a specific solution system (Schiller, 1991). However, no obvious differences in viscosity were observed among Matrix 1, Matrix 2, and Matrix 3 (Supplementary Figure S13). These results suggest that adding EP to the solutions change the thicknesses of the double layers at the liquid–gas interfaces.

For *α-*ionone, linalool oxide (furan), *β*-ionone, *cis*-theaspirane, dihydro-*β*-ionone, *trans*-theaspirane, linalool oxide (pyran), and *γ*-decalactone, the values of K increase after adding EP. This indicates that a reduction in the double-layer thickness facilitates faster interfacial mass transfer. For linalool, the value of K did not change obviously upon addition of EP; a reasonable explanation for this is that the high volatility of linalool diminishes the impact of EP on its release.

The unique structure of EP may contribute to the alteration of K; EP is an LCE with a hydrophobic long hydrocarbon group. The characteristic surfactant structure of EP causes its automatic congregation at the liquid–gas interface through molecular self-assembly (Conner et al., 1998). Once added to a solution, the molecular self-assembly of EP reduces the thickness of the double layer. However, the value of K did not increase continuously with further addition of EP, indicating that there were some stronger molecular interactions between the aroma compounds and EP, such as dipole–dipole interactions.

#### 3.2.3 Effects of EP on the aroma release amounts

Supplementary Table S4 shows the HC of each compound with EP after reaching the liquid–gas distribution equilibrium. The data show that the equilibrium HCs of the compounds decrease with increasing amounts of added EP, except for linalool and *β*-ionone. These results indicate that EP could affect the K_lg_ of these compounds, further suggesting that effective interactions exist between the aroma compounds and EP in the solutions.

**FIGURE 3:**
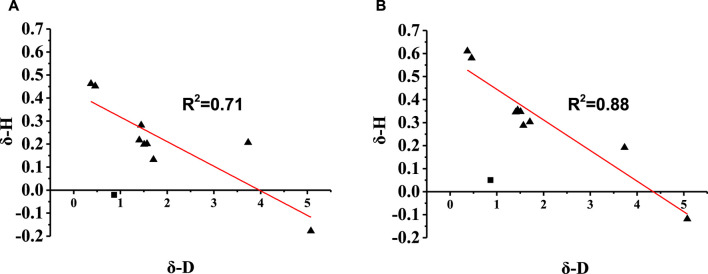
Relationship between the change ratio of the equilibrium HC (*δ*-H) and absolute dipole difference (*δ*-D) of the aroma compound with ethyl palmitate (EP) in **(A)** Matrix 2 and **(B)** Matrix 3.

The dipole moments of the aroma compounds and EP were calculated using Gaussian 09 and are listed in Supplementary Table S4. Figure 3 illustrates the relationship between the change ratio of the equilibrium HC (*δ*-H) and absolute dipole difference (*δ*-D) of the aroma compound with EP. The results show that the *δ*-H of the compounds have negative correlations with *δ*-D; this implies that when the dipole moment of EP is closer to that of the aroma compound, it has a greater effect on the release of the compound. Moreover, most compounds show linear correlations between *δ*-H and *δ*-D; this indicates that in the proposed solvent system, the intermolecular interactions between hexadecyl ethyl ester and the aromatic substances are mainly dipole interactions.

Therefore, it is a reasonable conclusion that dipole–dipole interactions are the main reason that EP affects the HC of the aroma compounds in the system comprising propylene glycol and ethanol. The effects of the dipole–dipole interaction are further demonstrated by the influence of EP on the release of the isomers. For theaspirane and linalool oxide (furan), the *δ*-H of the *cis*-isomer was higher than that of the *trans*-isomer, as these two compounds are enantiomers with different affinities to EP. These results are consistent with those reported by Honda et al. (2021).

#### 3.2.4 Effects of EP on the aroma release of the RO

To further evaluate the impacts of EP on the release of aroma compounds, the ODTs of the 10 compounds in Matrix 1, Matrix 2, and Matrix 3 were obtained using the 3-AFC and BET methods. As shown in Table 2, the ODT values of most compounds increased with the addition of EP. These results correspond with the decreased equilibrium HCs of the aroma compounds with the addition of EP, further verifying that EP could reduce the headspace release of the aroma compounds in solution. Linalool was still an exception in this case, and its ODT did not change significantly in different solvent media; this result is consistent with the release data of the compound.

**TABLE 2:** Thresholds of the aroma compounds in Matrix 1, Matrix 2, and Matrix 3.

Compound	Threshold (μg/g)		
	Matrix 1	Matrix 2	Matrix 3
*β*-Ionone	0.88	1.77	3.55
*γ*-Decalactone	0.22	0.44	0.88
*α*-Ionone	0.88	1.77	7.07
Dihydro-*β*-ionone	7.07	0.88	3.55
Linalool oxide (furan)	353.55	707.11	1414.21
Theaspirane	0.88	3.55	7.07
Linalool	0.088	0.088	0.088
Linalool oxide (pyran)	1767.77	3535.53	7071.07

**FIGURE 4:**
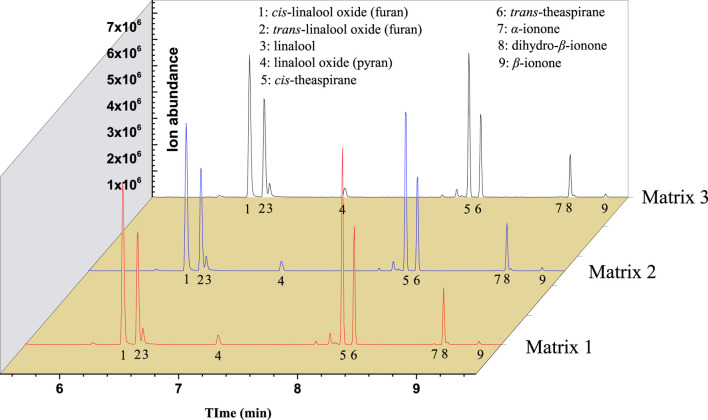
Headspace gas chromatography mass spectrometry (HS-GC/MS) TIC of reconstituted oil (RO) with different amounts of added ethyl palmitate (EP).

ROs with Osmanthus flavor were also prepared for the 10 selected aroma compounds according to their content in OFFA. These ROs were dissolved in Matrix 1, Matrix 2, and Matrix 3, and the equilibrium HCs were measured, as shown in Figure 4. The results indicate that among these compounds, the equilibrium HC values decrease with increasing EP content in the matrix.

**FIGURE 5:**
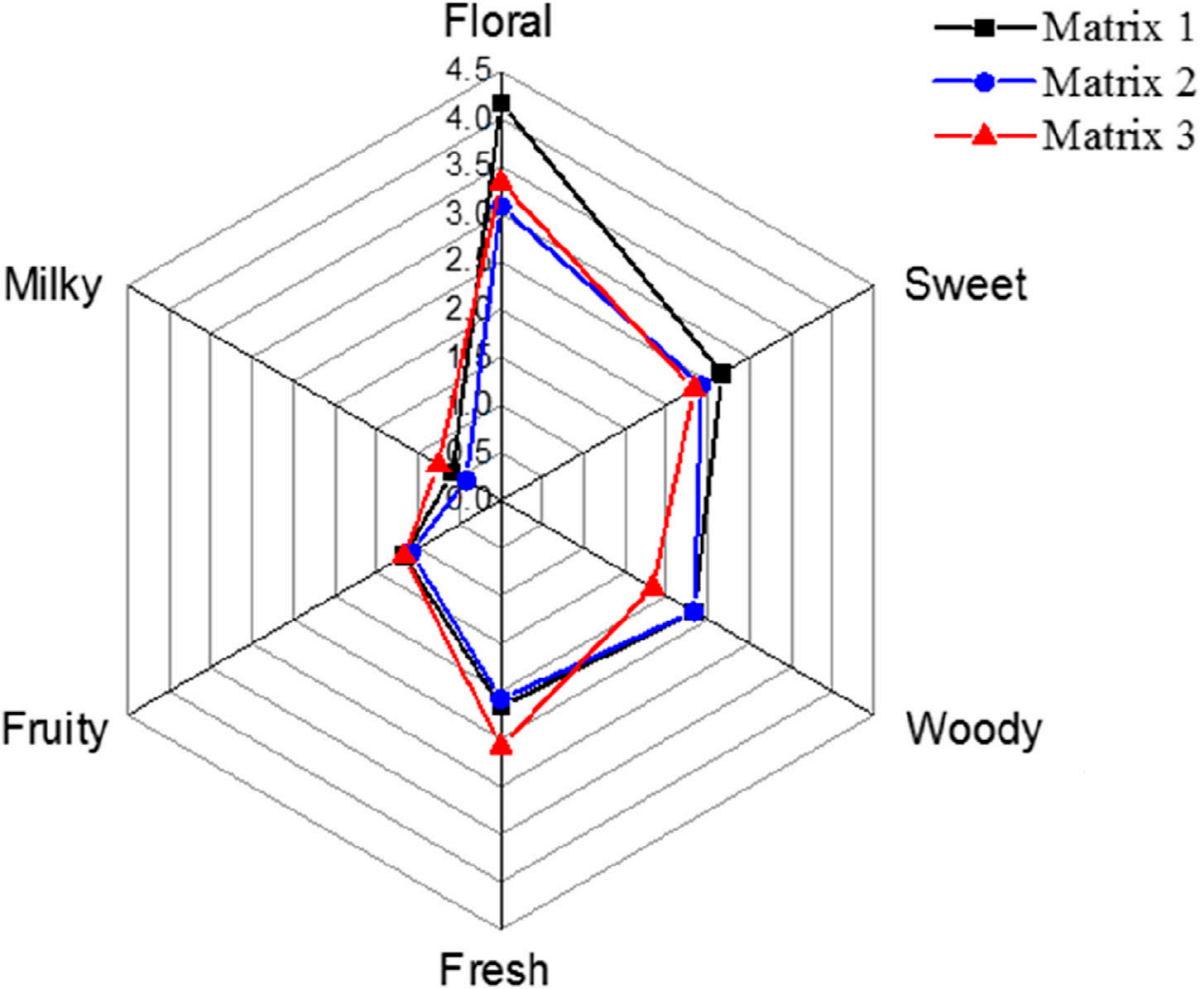
Aroma profiles of the ROs in different matrices.

The aroma profiles of the ROs in different media were also evaluated by experienced panelists using selected aroma descriptors, namely floral, sweet, woody, fresh, fruity, and milky, which were considered as the main flavors of OFFA. These results are depicted in Figure 5 and show that EP could change the score of each scent. The score variations were roughly correlated with the HCs of each of the compounds. In addition, EP decreased the odor intensities of the ROs and optimized the odors of the mixtures. This result further demonstrates that LCEs like EP could decrease the release of aroma compounds and also prolong the retention of the target fragrance.

## 4 Conclusion

The release characteristics of 10 aroma compounds from OFFA and their mixtures in the presence of EP in a solvent containing 1,2-propanediol and ethanol were obtained by HS-GC/MS and olfactometry. The results illustrate that EP reduces the equilibrium HCs of the selected compounds, increases their ODTs, and optimizes the odors of the solutions. Adding EP to RO reduces the HCs of the flavor compounds, indicating that EP plays an important role in the scent formation of OFFA. The experimental results of static and dynamic headspaces show that EP changes the K and K_lg_ of the aroma compounds between the liquid and gas phases. These release kinetics could be explained by Whitman’s two-film model. Accordingly, the mass transfer is faster between the liquid and gas phases. The *δ*-H of most selected compounds show negative correlations with their *δ*-D values, indicating that dipole–dipole interactions of the volatiles and EP is the important factor affecting K_lg_. These research findings significantly enhance our understanding of the role of EP in modulating the release of aromatic compounds. Further, these insights are pivotal in guiding the development of new perfume formulations with enhanced sensory properties.

## Data Availability

The original contributions presented in the study are included in the article/Supplementary Material, and any further inquiries may be directed to the corresponding author.
