# Supplementary material for: Effects of ethyl palmitate on the release of aroma compounds in propanediol–ethanol solution and its mechanisms
Source: Front Chem. 2024 Jun 10;12:1381835. doi: 10.3389/fchem.2024.1381835 (PMC11194716; doi:10.3389/fchem.2024.1381835)
Supplement: Supplementary file 1 [file DataSheet1.docx]

Supplementary Material

# Supplementary Figures and Tables

## Supplementary Figures

**Supplementary** **Figure 1.** The TIC of Osmanthus fragrans flower absolute (OFFA)

**Supplementary Figure 2.** The TIC of OFFA with different EP addition

**The explain of aroma release by Whitman’s double-layer model:**

Whitman’s double-layer model was illustrated as equation (S1):

$C_{hs}\left( t \right)=K_{lg}C_{bp}(1-exp[-\left( \frac{KA}{V} \right)t])$ Equation (S1)

The fitting process of release curves of aroma compounds was conducted by OriginPro9. Fitting model BoxLucas-1 was selected as the working model. In Boxlucas-1 model, equation (S2) was used to fit the scatter of headspace concentration (HC)-time.

$y=a*(1-\exp\left( -bx \right))$ Equation (S2)

Comparing equation (S1) and (S2), the parameter a and b of equation (S2) were defined as: a = K_lg_C_bp_ and b = KA/V. The parameter a was proportional to K_lg_ and parameter b was proportional to A/V. Therefore, the change of parameter a and b could reflect the change of K_lg_ and K in a certain aroma release system.

**Supplementary Figure 3.** The release curve of cis-linalool oxide (furan) in Matrix 1, Matrix 2 and Matrix 3.

**Supplementary Figure 4.** The release curve of trans-linalool oxide (furan) in Matrix 1, Matrix 2 and Matrix 3.

**Supplementary Figure 5.** The release curve of dihydro-β-ionone in Matrix 1, Matrix 2 and Matrix 3.

**Supplementary Figure 6.** The release curve of cis-theaspirane in Matrix 1, Matrix 2 and Matrix 3.

**Supplementary Figure 7.** The release curve of trans-theaspirane in Matrix 1, Matrix 2 and Matrix 3.

**Supplementary Figure 8.** The release curve of β-ionone in Matrix 1, Matrix 2 and Matrix 3.

**Supplementary Figure 9.** The release curve of α-ionone in Matrix 1, Matrix 2 and Matrix 3.

**Supplementary Figure 10.** The release curve of linalool oxide (pyran) in Matrix 1, Matrix 2 and Matrix 3.

**Supplementary Figure 11.** The release curve of γ-decalactone in Matrix 1, Matrix 2 and Matrix 3.

**Supplementary Figure 12.** The release curve of linalool in Matrix 1, Matrix 2 and Matrix 3.

**The apparent shear viscosity:**

The apparent shear viscosity of Matrix 1, Matrix 2 and Matrix 3 was measured by a microfluidic viscometer (RheoSense Inc., U.S.A.), the operator parameters were as follows: Shear Rate: 1000 s^-1^ Measurement Time: 65 s; Waiting Time:15 s; Temperature: 25 °C.

**Supplementary Figure 13. The apparent shear viscosity of Matrix 1, Matrix 2 and Matrix 3.**

## Supplementary Tables

**Supplementary Table 1.** Chemical composition of OFFA.

| Peak | R. T.(min) | Percent of peak area (%) | Compound | Molecular weight |
| --- | --- | --- | --- | --- |
| 1 | 9.007 | 0.795 | 2-methylcyclopentanone | 98.14 |
| 2 | 11.923 | 4.812 | *cis*-linalool oxide (furan) | 170.25 |
| 3 | 12.178 | 7.205 | *trans*-linalool oxide (furan) | 170.25 |
| 4 | 12.289 | 1.369 | linalool | 154.25 |
| 5 | 13.56 | 6.233 | linalool oxide (pyran) | 170.25 |
| 6 | 15.043 | 1.378 | 1-phenylethyl propionate | 178.23 |
| 7 | 15.605 | 0.981 | *cis*-theaspirane | 194.31 |
| 8 | 15.807 | 1.269 | *trans*-theaspirane | 194.31 |
| 9 | 16.424 | 2.113 | 4-methoxyphenethyl alcohol | 152.19 |
| 10 | 17.151 | 0.403 | *α*-ionone | 192.3 |
| 11 | 17.315 | 9.051 | dihydro-*β*-ionone | 194.31 |
| 12 | 17.426 | 5.362 | dihydro-*β*-ionol | 196.33 |
| 13 | 17.693 | 2.247 | *γ*-decalactone | 170.25 |
| 14 | 17.9 | 3.775 | *β*-ionone | 192.3 |
| 15 | 18.014 | 1.113 | 4-hexyloxyaniline | 193.29 |
| 16 | 18.905 | 1.141 | DL-Menthone | 154.25 |
| 17 | 19.101 | 1.009 | piperonone | 152.23 |
| 18 | 20.268 | 2.69 | 3-methyl-5-nitrosotolone | 165.04 |
| 19 | 20.398 | 1.208 | 3-oxodihydro-*β*-ionone | 208.15 |
| 20 | 20.801 | 3.115 | 3-oxodihydro-*β*- ionol | 210.16 |
| 21 | 23.052 | 5.297 | palmitic acid | 256.42 |
| 22 | 23.379 | 3.939 | ethyl palmitate | 284.48 |
| 23 | 24.744 | 2.64 | (*Z, Z*)-9,12-octadecadienoic acid | 280.45 |
| 24 | 24.813 | 12.149 | (*Z*, *Z*, *Z*)-9,12,15-octadecatrienoic acid | 278.43 |
| 25 | 24.999 | 4.409 | (*Z*)-9,17-octadecadienal | 264.45 |
| 26 | 25.065 | 8.554 | 8,11,14-eicosatrienoic acid methyl ester | 320.51 |
| 27 | 26.18 | 0.76 | 3-methylheptadecane | 254.49 |
| 28 | 27.782 | 0.93 | 1-nonadecene | 266.51 |
| 29 | 29.886 | 4.056 | eicosan | 282.55 |

**Supplementary Table 2.** Linear equation, correlation coefficient (R2), concentrations of the selected components in OFFA.

| Compounds | Linear equation | *R*^2^ | Concentration(mg/g) |
| --- | --- | --- | --- |
| *cis*-linalool oxide (furan) | *y* = 0.897*x*+0.071 | 0.999 | 14.4±0.41 |
| *trans*-linalool oxide (furan) | *y*=0.743*x*+0.108 | 0.999 | 17.8±0.46 |
| linalool | *y*=0.590*x*-0.061 | 1 | 3.52±0.11 |
| linalool oxide (pyran) | *y*=0.668*x*+0.041 | 0.999 | 17.21±0.54 |
| *cis*-theaspirane | *y*=1.491*x*-0.103 | 0.999 | 4.53±0.19 |
| *trans*-theaspirane | *y*=1.507*x*-0.432 | 0.999 | 4.24±0.17 |
| *α*-ionone | *y*=0.983*x*-0.075 | 0.999 | 1.10±0.03 |
| dihydro-*β*-ionone | *y*=0.625*x*+0.017 | 0.999 | 22.49±0.24 |
| *γ*-decalactone | *y*=1.303*x*+0.470 | 0.999 | 6.25±0.30 |
| *β*-ionone | *y*=1.073*x*+0.283 | 1 | 10.29±0.42 |

**Supplementary Table 3.** The ingredients of the reconstituted oil (RO).

| Compounds | Percent of weight |
| --- | --- |
| *β*-ionone | 9.82% |
| *α*-ionone | 1.05% |
| *γ*-decalactone | 23.53% |
| dihydro-*β*-ionone | 31.22% |
| linalool oxide (furan) (isomer mixture) | 16.20% |
| linalool oxide (pyran) | 8.80% |
| Theaspirane (isomer mixture) | 3.56% |
| linalool | 9.82% |

**Supplementary Table 4.** The equilibrium HCs and dipole moments of 10 aroma compounds and EP.

| Compounds | equilibrium HC | | | dipole |
| --- | --- | --- | --- | --- |
|  | Matrix 1 | Matrix 2 | Matrix 3 |  |
| *β*-ionone | 98278 | 110475 | 114180 | 7.658789 D |
| *γ*-decalactone | 11675 | 9721 | 9377 | 6.315365 D |
| *α*-ionone | 264436 | 220709 | 187739 | 4.288903 D |
| dihydro-*β*-ionone | 1.05ⅹ10^7^ | 8.00ⅹ10^6^ | 6.47ⅹ10^6^ | 4.090773 D |
| *cis*-linalool oxide (furan) | 4.37ⅹ10^7^ | 3.27ⅹ10^7^ | 2.79ⅹ10^7^ | 4.028002 D |
| *trans*-linalool oxide (furan) | 3.09ⅹ10^7^ | 2.38ⅹ10^7^ | 2.02ⅹ10^7^ | 3.986402 D |
| ethyl palmitate | / | / | / | 2.582366 D |
| *trans*-theaspirane | 1.47ⅹ10^7^ | 8.31ⅹ10^6^ | 6.56ⅹ10^6^ | 2.213651 D |
| *cis*-theaspirane | 2.27ⅹ10^7^ | 1.19ⅹ10^7^ | 9.28ⅹ10^6^ | 1.726123 D |
| linalool | 1.11ⅹ10^7^ | 1.02ⅹ10^7^ | 1.00ⅹ10^7^ | 1.015138 D |
| linalool oxide (pyran) | 6.81ⅹ10^7^ | 5.51ⅹ10^7^ | 4.72ⅹ10^7^ | 0.901738 D |
