# Supplementary material for: Effects of ethyl palmitate on the release of aroma compounds in propanediol–ethanol solution and its mechanisms
Source: Front Chem. 2024 Jun 10;12:1381835. doi: 10.3389/fchem.2024.1381835 (PMC11194716; doi:10.3389/fchem.2024.1381835)
Supplement: Supplementary file 2 [file Image1.pdf]

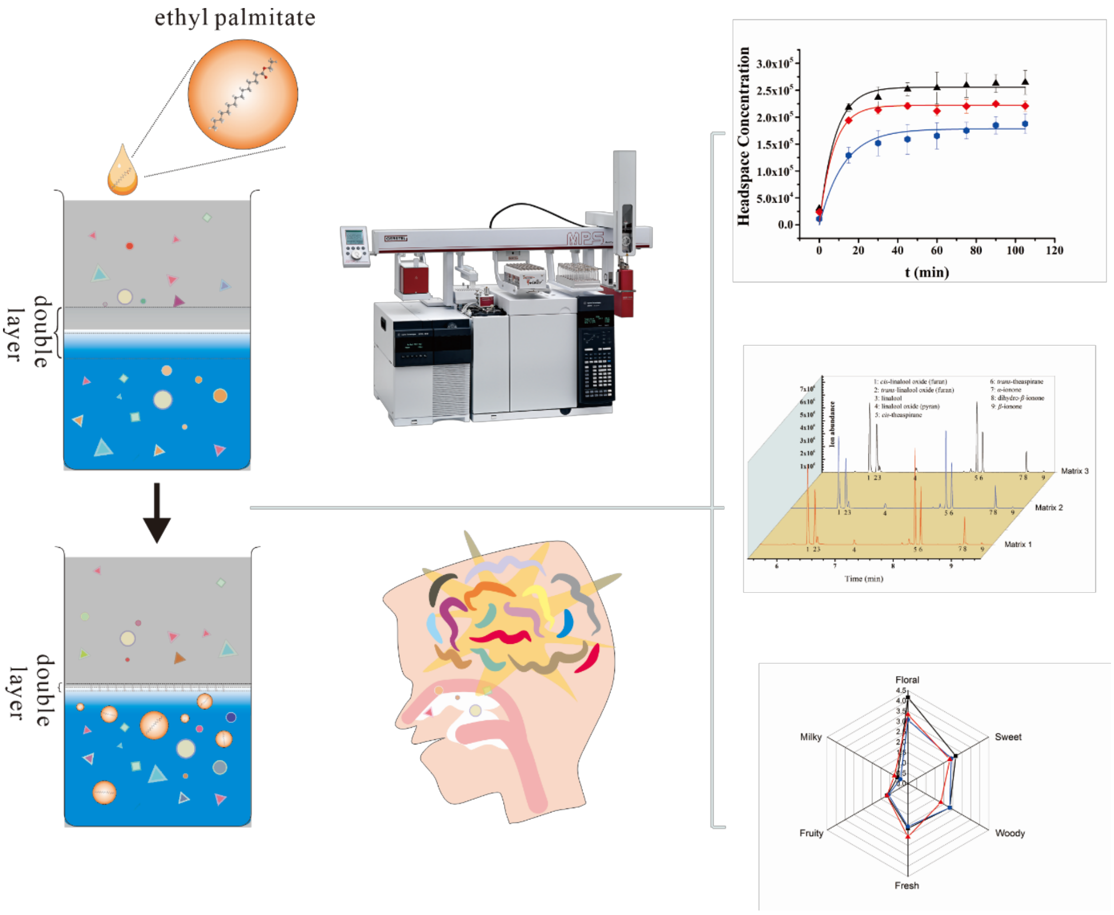

EP affects the release behavior of aroma compounds by self-assemble effect and dipole-dipole interaction.
